# Immune Thrombocytopenia in a Very Elderly Patient With Covid-19

**DOI:** 10.3389/fmed.2020.00404

**Published:** 2020-07-10

**Authors:** Fehmi Hindilerden, Ipek Yonal-Hindilerden, Senoglu Sevtap, Kadriye Kart-Yasar

**Affiliations:** ^1^Hematology Clinic, Bakirköy Dr. Sadi Konuk Training and Research Hospital, University of Health Sciences, Istanbul, Turkey; ^2^Division of Hematology, Department of Internal Medicine, Istanbul Medical Faculty, Istanbul University, Istanbul, Turkey; ^3^Department of Microbiology and Infectious Diseases, Bakirköy Dr. Sadi Konuk Training and Research Hospital, University of Health Sciences, Istanbul, Turkey

**Keywords:** immune thrombocytopenia, Covid-19, very old age, intravenous immunoglobulin, corticosteroids

## Abstract

Immune thrombocytopenia (ITP) is an autoimmune disorder characterized by a decreased number of platelets and mucocutaneous bleeding. Many viruses have been identified as triggers of the autoimmune process, including human immunodeficiency virus (HIV), hepatitis C virus (HCV), Epstein-Barr virus (EBV), cytomegalovirus (CMV), parvovirus, rubella, and measles. Association with the new severe acute respiratory syndrome coronavirus, SARS-CoV-2 infection (Covid-19 infection) has been rarely reported. Here, we report the oldest case of ITP patient triggered by the novel coronavirus infection. He showed inadequate response to IVIG but responded to corticosteroids with no severe adverse events. Further studies are warranted to determine the optimal therapeutic strategies for ITP with the Covid-19 infection.

## Introduction

Immune thrombocytopenia (ITP) is a rare autoantibody-mediated disorder characterized by a platelet count of <100,000/mm^3^, mostly with minor mucosal bleeding (1). ITP occurs either *de novo* or secondary to other underlying disorders. Common conditions associated with secondary ITP include lymphoproliferative disorders, other autoimmune disorders and collagen vascular diseases. ITP is also associated with certain, mostly viral, infections (2). HIV and HCV are well-characterized causes of ITP while Epstein-Barr virus (EBV), cytomegalovirus (CMV), herpes viruses, parvovirus, rubella, and measles have also been identified as causes of ITP (2). An ongoing outbreak of SARS-CoV-2 infection (Covid-19 infection) was first identified in Wuhan, Hubei province, China in December 2019 (3). Thrombocytopenia is a risk factor for increased morbidity and mortality in patients with Covid-19 infection (4). Thrombocytopenia in Covid-19 patients may be the result of disseminated intravascular coagulation (DIC), sepsis or may be drug-induced (4). ITP in Covid-19 has been rarely reported (5, 6). We report a 86-year-old male diagnosed with Covid-19 infection, who at initial diagnosis of Covid-19 infection presented with severe ITP.

## Case Presentation

**Figure 1:**
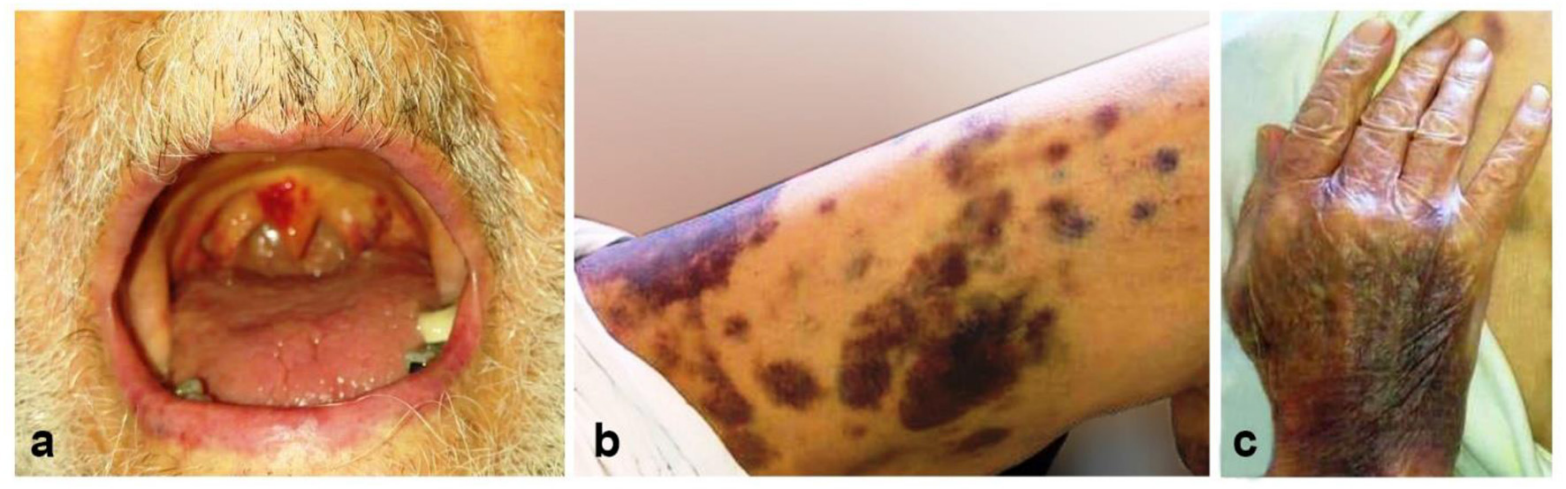
The appearance of the lesions at admission to our center. Hemorrhagic bullous lesions in the oral cavity **(a)**, ecchymotic and purpuric lesions scattered over the lower extremity **(b)**, and dorsum of the hand **(c)**.

**Figure 2:**
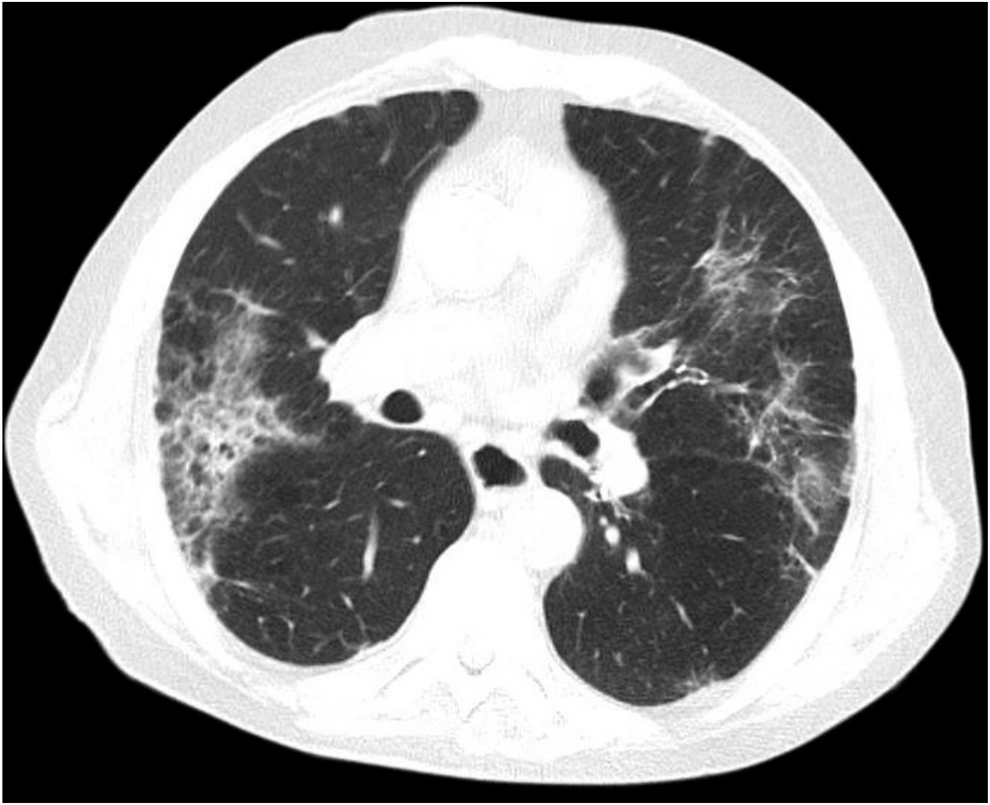
Chest computed tomography shows widespread scattered ground-glass opacities in both lungs, findings compatible with severe Covid-19 pneumonia.

A 86-year-old man with a history of hypertension and type 2 diabetes presented with a 1-week history of excessive bruising, fatigue, fever, and dry cough. He had known Covid-19 exposure. On physical examination, he was subfebrile (37.6°C) and had a respiratory rate of 24/min. There were purpuric eruptions widely scattered over the skin and hemorrhagic bullae in the oral cavity (Figure 1). The patient's clinical signs included hypoxaemia (pulse oximetry 91% on ambient air) and sinus tachycardia (110 beats/minute). Lung auscultation revealed diminished breath sounds with fine bibasilar crackles. The laboratory tests showed the following: hemoglobin 11 g/dL, total leukocyte count 4,020/mm^3^, neutrophil 2,930/mm^3^, lymphocyte: 960/mm^3^, and platelet count 10,000/mm^3^ (Table 1). On biochemical tests, C-reactive protein was elevated at 15 mg/L (normal range, 0–5) with normal procalcitonin level (0.07 ng/ml; normal range < 0.5). Serum ferritin, LDH, and Troponin-I levels were normal (65 μg/ml, 247 U/L, and 10 pg/ml, respectively). Prothrombin and activated partial thromboplastin time were normal. Fibrinogen level was 379 mg/dl (normal range, 200–400) and D-Dimer was slightly elevated (0.97 μg/ml; normal range, 0–0.5) (Table 1). Reverse transcriptase PCR assay detected the presence of SARS-CoV-2 RNA in the nasopharyngeal swab. Chest computed tomography (CT) showed widespread scattered ground-glass opacities in both lungs, findings compatible with severe Covid-19 pneumonia (Figure 2). On peripheral blood smear, there were no schistocytes or atypical cells. Peripheral blood confirmed the presence of thrombocytopenia. Given the very old age of the patient and a possible association with multiple myeloma (MM) and ITP, immunoglobulin levels, serum and urine immunofixation and protein electrophoresis were checked and all were found to be within normal ranges (7). Bone marrow aspiration revealed a normocellular bone marrow with concomitant increase in normal sized megakaryocytes. The other cell lines were normal and there was no sign of dysplasia and hemophagocytosis. Bone marrow biopsy also revealed increased number of megakaryocytes in the absence of other significant abnormalities. The cytogenetic analysis revealed normal karyotype. To treat Covid-19 pneumonia, favirapivir 1,600 mg twice daily on day 1, followed by 600 mg twice daily for a total duration of 5 days and azithromycin 500 mg on day 1 plus 250 mg daily on days 2–5 were started. It was considered that the patient developed secondary ITP triggered by Covid-19. Other viral, autoimmune and malignant diseases were screened and found to be negative. Due to the presence of hemorrhagic bullae, the patient was regarded to have high risk for life threatening bleeding due to secondary ITP. Intravenous immunoglobulin (IVIG) was administered at a rate of 1 g/kg body weight for two consecutive days. Three days after the initiation of IVIG, his platelet count was 25,000/mm^3^. Thus, oral prednisolone at a dose of 1 mg/kg/day was started. On the 10th day of admission to hospital, the purpura had disappeared and his oxygen saturation on ambient air was 96%. His platelet count increased to 100,000/mm^3^. Due to the hematological response attained on the 7th day of prednisolone and taking into consideration the side effects of corticosteroids related to his comorbidities and very old age, the dose was decreased to 0.5 gr/kg/day and planned to be stopped in 4 weeks. He is now on the 3th week of the corticosteroid treatment at a dosage of 0.25 gr/kg/day with no bleeding symptoms. The final laboratory tests showed the following: hemoglobin 11 g/dL, total leukocyte count 4,200/mm^3^, neutrophil 2,400/mm^3^, lymphocyte: 1,680/mm^3^ and platelet count 150,000/mm^3^.

**Table 1:** Characteristics of the patient with Covid-19 associated ITP.

	**Patient**
Age	86
Gender	Male
Hemoglobin level (g/dL)	11
Leukocyte level (/mm^3^)	4,020
Lymphocyte level (/mm^3^)	960
Platelet count (/mm^3^)	10,000
C-reactive protein (mg/L)	15
Procalcitonin level (ng/ml)	0.07
Serum ferritin (μg/ml)	65
LDH level (U/L)	247
Troponin-I (pg/ml)	10
Fibrinogen level(mg/dl)	379
D-dimer(μg/ml)	0.97
Chest CT	Severe pneumonia
Nasopharyngeal swab (tested by PCR)	Positive

## Discussion

Covid-19 is a systemic infection with significant impact on the hematopoietic system. On admission, 36.2% of patients present with thrombocytopenia, which is more prominent among severe vs. non-severe cases (57.7 vs. 31.6%) (8). Thrombocytopenia is a risk factor for increased morbidity and mortality in Covid-19 infection %) (4). Thrombocytopenia in Covid-19 patients may be caused by sepsis, disseminated intravascular coagulation (DIC), or drug-induced (4). Recently, several case reports have suggested that ITP may be associated with Covid-19 infection (5, 6). ITP is a rare autoimmune disease, in which with many viruses including mainly HIV and HCV have been identified as triggers of the autoimmune process (2).

The mechanism of virus-induced thrombocytopenia has not been clearly elucidated. Viruses may cause a decrease in platelet production by infecting megakaryocytes. This results in apoptosis of megakaryocytes, decreased maturation of megakaryocytes or decreased expression of the thrombopoietin receptor. Viruses may also infect hematopoietic stem cells and result in a decrease of progenitor cells and induction of growth deficient megakaryocyte colony forming units, due to disordered production of cytokines by the infected cells in the bone marrow. Another proposal for virus induced thrombocytopenia is by platelet destruction where viruses either directly interact with platelets or recognize immunocomplexes of IgGs and viral antigens (9). Bone marrow examination of our patient showed no suppression of the hematopoietic precursors but an increase in the number of megakaryocytes suggesting that there was immune mediated destruction of platelets. The precise mechanism of ITP associated with the novel coronavirus has not been clearly elucidated. It is presumed that following the infection, the immune responses raised against Covid-19 may cross-react with human proteins that share peptide sequences with the virus and thus result in autoimmune pathologic sequelae (10). Zulfiqar et al. was the first to report ITP in a 65-year-old patient with Covid-19 (5). In that report, ITP developed after cessation of Covid-19 associated symptoms. Bomhof et al. reported a case series of ITP in Covid-19 patients including a 67-year-old man, a 66-year-old woman and a 59-year-old man (6). In the aforementioned case series, ITP developed not only during active COVID-19 infection, but also up to 10 days after the resolution of Covid-19 symptoms. Our patient developed ITP at initial presentation as he was suffering from Covid-19 associated symptoms. First reported case of ITP in the course of Covid-19 responded to prednisolone and eltrombopag (5). While two of the three reported cases recovered from ITP with IVIG and dexamethasone, one patient died of intracerebral bleeding because of delay in diagnosis (6).

To our knowledge, our case is the oldest case of ITP patient triggered by Covid-19 infection. Studies of older ITP adults are lacking, and recommendations for management are based mainly on expert opinion. The treatment of ITP may be difficult, especially in patients older than 75 years (very old age) and must take into account the comorbidities, concurrent medications and severity of bleeding. The mechanism of increased risk of bleeding in older age ITP patients are not at all completely understood, but age is associated with endothelial dysfunction (11). Herein we reported a very old Covid-19 patient presenting with hemorrhagic bullous lesions with high propensity to life threatening bleeding. With prolonged life expectancy, the frequency of ITP has increased and become more challenging in the elderly. Yet, our case implies that IVIG and corticosteroids may remain as optimal first-line treatments in elderly Covid-19 patients presenting with ITP. Eltrombopag is commonly used for treatment of ITP (12). Since eltrombopag, in selected cases, have posed an increased risk for venous thromboembolism, it should be used with caution in Covid-19 infection, which itself is reported to result in a hypercoaguable state (13, 14). Furthermore, there is limited data on the safety of eltrombopag in older ITP patients. The incidence of a thrombotic event was significantly increased in ITP patients ≥65 years undergoing eltrombopag treatment (15). After inadequate response to IVIG, our patient received prednisolone resulting in a platelet count of 100,000/mm^3^ on day 10. We refrained from the use of eltrombopag because of the lack of safety data in the elderly ITP patients and to avoid the risk of exacerbation of coagulation activation by Covid-19 infection.

Herein, we reported a case of a 86-year-old male patient with a background history of hypertension, type 2 diabetes and a positive swab for Covid-19, who presented with excessive bruising, fatigue, fever, and dry cough and signs of pneumonia. Our patient had no history of autoimmune disorder. The distinctive feature of our patient is his very old age and that he develops ITP at initial presentation as he was suffering from Covid-19 associated symptoms not after the resolution of Covid-19 symptoms, which contrasts with most of the previous cases reporting ITP after the resolution of Covid-19 symptoms (5, 6). Our findings support that ITP at initial presentation may be observed in Covid-19 infected patients and other potential causes of thrombocytopenias should be excluded in these patients to avoid lethal complications and deliver appropriate treatment. Given the efficacy of steroids and IVIG based on expert opinion in elderly ITP patients, this therapy is worth considering as a treatment of elderly ITP with the Covid-19 infection. However, taking into account the report by “Centers for Disease Control and Prevention” and “World Health Organization” that states corticosteroids may inhibit immune responses and pathogen clearance of Covid-19, prolonged treatment with steroids should be avoided.

## Data Availability Statement

All datasets presented in this study are included in the article/supplementary material.

## Ethics Statement

The studies involving human participants were reviewed and approved by Ethics Comittee of Bakirköy Dr. Sadi Konuk Training and Research Hospital. The patients/participants provided their written informed consent to participate in this study. Written informed consent was obtained from the individual(s) for the publication of any potentially identifiable images or data included in this article.

## Author Contributions

All authors collected data, wrote, and revised the article.

## Conflict of Interest

The authors declare that the research was conducted in the absence of any commercial or financial relationships that could be construed as a potential conflict of interest.
